# Carpal Tunnel Syndrome in the Very Elderly: Clinical, Electrodiagnostic, and Ultrasound Features in a Cohort of 187 Patients

**DOI:** 10.3390/neurolint17090137

**Published:** 2025-08-30

**Authors:** Lisa B. E. Shields, Vasudeva G. Iyer, Theresa Kluthe, Yi Ping Zhang, Christopher B. Shields

**Affiliations:** 1 Norton Neuroscience Institute, Norton Healthcare, 210 East Gray Street, Suite 1102, Louisville, KY 40202, USA; lbes@earthlink.net (L.B.E.S.); yipingzhang50@gmail.com (Y.P.Z.); 2 Neurodiagnostic Center of Louisville, Louisville, KY 40245, USA; pavaiyer@gmail.com; 3 Norton Research Institute, Norton Healthcare, Louisville, KY 40202, USA; theresa.kluthe@louisville.edu; 4 Department of Pediatrics, University of Louisville School of Medicine, Louisville, KY 40202, USA

**Keywords:** carpal tunnel syndrome, elderly, electrodiagnostic study, ultrasonography

## Abstract

Background/Objectives: Elderly patients with carpal tunnel syndrome (CTS) have more severe clinical, ultrasonic, and electrodiagnostic (EDX) findings compared to younger patients. Thenar weakness and atrophy are more common at initial presentation in the elderly population with CTS. Methods: This is a retrospective review of 187 very elderly patients (aged 80 years and older) with EDX confirmation of CTS. We describe the clinical, EDX, and US features in these patients and compare the severity of the median nerve entrapment at the carpal tunnel (CT) by EDX findings to a middle-aged cohort (ages 40–50 years). Results: The total number of very elderly hands with CTS was 289 (187 patients total, with bilateral symptoms in 102 patients). Of the 289 hands, thenar atrophy was observed in 75 (26.0%) hands, weakness of the abductor pollicis brevis (APB) muscle was detected in 178 (61.6%) hands, and pinprick decrease/loss was noted in 265 (91.7%) hands. Of the total 289 hands, 57 (66.3%) hands’ median nerve stimulation did not evoke compound muscle action potentials over the APB and second lumbrical muscles. Sensory nerve action potentials were not detected in 211 (76.2%) hands. Comparing the sensitivities of various US measurements in diagnosing CTS, the cross-sectional area at the CT inlet had the highest sensitivity among the various measurements. As the CSA at the CT inlet increases, the odds of a greater CTS severity by EDX studies also increase (OR = 1.109, *p*-value = 0.001). The very elderly patients with CTS more frequently had more severe CTS compared to the middle-aged patients with CTS (chi-squared = 102.65_3_, *p*-value < 0.001). Conclusions: The very elderly patients appear to seek medical care only when the CTS has become severe. The primary care physicians should look for signs and symptoms of CTS in the very elderly and encourage prompt treatment. Surgeons should be cognizant of the differences in the clinical, EDX, and US studies in the very elderly patient cohort with CTS. US is highly useful in evaluating CTS when the EDX studies become non-localizing in severe CTS, as often seen in the very elderly patients.

## 1. Introduction

Carpal tunnel syndrome (CTS) is the most frequent focal mononeuropathy, encompassing 90% of all compressive neuropathic conditions [1]. The incidence of CTS is 1–3 cases per 1000 individuals annually in the United States, with a prevalence of approximately 50 cases per 1000 individuals [2]. This syndrome arises when the median nerve is compressed as it traverses through the carpal tunnel (CT) [1]. The diagnosis of CTS is based on characteristic clinical symptoms (paresthesia, pain, and muscle weakness in the median nerve distribution), clinical signs (hypoesthesia in the median nerve distribution and weakness of the thenar muscles), nerve conduction studies (measuring the extent of demyelination/conduction block in the median nerve and denervation of the thenar muscles), and ultrasound (US) findings. US is a simple, noninvasive, low-cost, rapid, and reliable option for confirming CTS, as it is easy to evaluate the median nerve within the CT [3,4,5,6,7,8]. A debate exists as to the best location to measure the median nerve and the specific threshold to confirm the diagnosis of CTS [7]. An increased median nerve cross-sectional area (CSA) at the wrist is reportedly diagnostic of CTS; however, there is variability in reported normal values [4,9]. The wrist-to-forearm ratio (WFR) of the median nerve CSA of ≥1.4 reportedly has a 100% sensitivity for identifying patients with CTS, while the median nerve CSA at the wrist has a sensitivity of 45–93% [4]. US is also valuable in determining the severity of CTS based on the CSA of the median nerve at the wrist. According to one study, patients with a median nerve CSA of 14 mm^2^ or more have a very high probability of moderate to severe CTS [8].

It has been suggested that a bimodal age distribution exists for CTS, with the initial peak between 50 and 54 years and the second peak between 75 and 84 years [10]. Several studies have evaluated CTS in older adults compared to younger patients [9,11,12,13,14]. Certain characteristics are more common in the elderly age group, including thenar muscle atrophy, diurnal paresthesia, and more severe motor and sensory loss [12,14,15]. The definition of “elderly” patients with CTS varies in the literature, with some studies labeling “elderly” as ≥65 years [12,16], ≥70 years [14,17], and ≥72 years [11]. The term “super-elderly” has referred to patients 80 years and older [15]. The greater severity of clinical, ultrasonic, and EDX findings may be due to age-related changes in the median nerve’s response to compression in the very elderly group with CTS compared to younger patients with CTS [17]. An age-related decline in collagen I expression in the transverse carpal ligament may also occur, which may lead to its degeneration. Additionally, slower axonal regeneration and decreased density of regenerating axons after nerve injury are often observed in very elderly patients with CTS compared to younger patients [17].

In this report, we evaluate the clinical, EDX, and US features of very elderly patients (ages 80 years and older) with CTS. We compare the severity of the median nerve entrapment at the CT by EDX findings between the very elderly cohort to a younger age cohort (ages 40–50 years). We also discuss why CTS may be more severe in the very elderly population.

## 2. Materials and Methods

**Figure 1:**
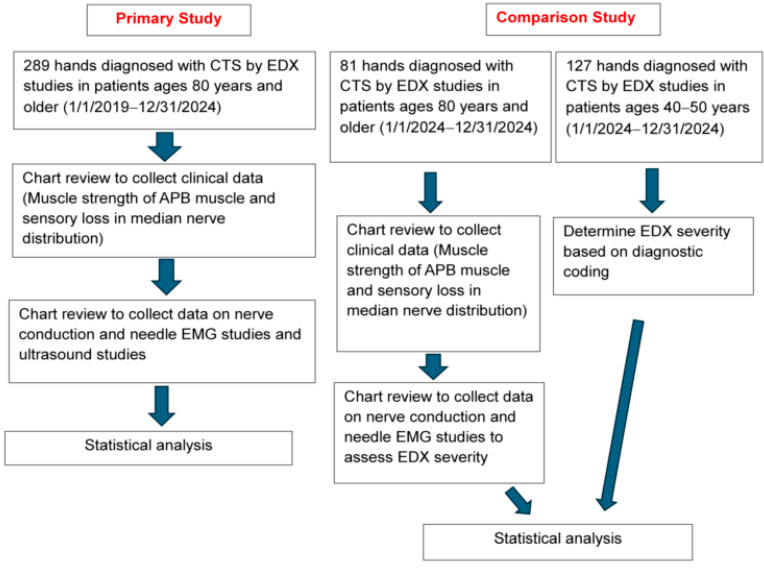
Flow chart of the study design, highlighting the primary study (hands of patients with carpal tunnel syndrome ages 80 years and older between 1 January 2019 and 31 December 2024) and the comparison study (comparing hands of patients ages 80 and older to patients ages 40–50 years between 1 January 2024 and 31 December 2024). The EDX severity for the middle-aged patients (ages 40–50 years) was based on diagnostic coding. CTS: carpal tunnel syndrome. EDX: electrodiagnostic. APB: abductor pollicis brevis.

Under an Institutional Review Board (IRB)-approved protocol, we performed a 6-year (1 January 2019–31 December 2024) retrospective analysis of very elderly patients (80 years and older) referred to our Neurodiagnostic Center for EDX studies to confirm the diagnosis of CTS. According to the American Geriatric Society and the World Health Organization, the “oldest-old” is defined as individuals aged over 80 years [18]. Therefore, we selected 80 years as the cutoff for the “very elderly” patients with CTS in the current study. An analysis was also performed comparing two groups of patients with CTS for a 1-year duration (1 January 2024–31 December 2024) at our Neurodiagnostic Center: (1) ages 80 years and older and (2) ages 40–50 years. Figure 1 features the flow chart of the study design. The severity of the median nerve entrapment at the CT by EDX findings was evaluated between these two age cohorts with CTS. We selected 2024 as the benchmark year for the comparison between the very elderly (ages 80 years and older) and middle-aged groups (ages 40–50 years) since 2024 is representative of the previous years with regard to the number of cases. The EDX severity for the middle-aged patients (ages 40–50 years) was based on diagnostic coding.

Our AANEM-accredited Neurodiagnostic Center evaluates approximately 1000 patients annually who are referred for EDX studies by a variety of physicians, including hand surgeons, neurosurgeons, orthopedic surgeons, neurologists, and rheumatologists. This center also receives referrals from family physicians, internists, and nurse practitioners. Patients are referred for a wide range of suspected diagnoses, from CTS to cervical radiculopathy to amyotrophic lateral sclerosis. Our independent Neurodiagnostic Center does not have any referral bias, and we evaluate all neuromuscular problems, not just CTS. Our protocol was for the electromyographer, a neurologist who is board-certified in electrodiagnostic medicine and clinical neurophysiology, to perform a focused neurological examination, including muscle strength, pinprick sensation, and reflexes in the upper extremities, followed by nerve conduction and EMG studies as well as ultrasonography.

The EDX protocol included both nerve conduction and needle EMG studies using the standard protocol in our lab [19]. Median nerve motor conduction velocity across the forearm and wrist was determined by placing the recording electrode over the abductor pollicis brevis (APB) muscle. When there was no CMAP over the APB muscle, the study was performed with the recording electrode over the second lumbrical muscle. Sensory conduction was studied with the recording electrode over digits 2 and 3 and antidromic stimulation at the wrist.

The US protocol included studying long and short-axis images of the median nerve at the wrist and the forearm, as well as of the thenar muscles, using an 8–18 or 6–15 MHz probe. We used 2 different US GE machines in our study depending upon availability, one that accommodated multiple probes (6–15 and 8–18 MHz) and the other only the 8–18 MHz probe. We adjusted the depth and frequency in each to obtain the best image quality. US studies documented the CSA at the CT inlet/outlet and forearm (CT inlet at the distal wrist crease and CT outlet 4 cm distal to the CT inlet in the proximal palm). The CSA is measured by the “trace” technique, and the higher of the two values (CT inlet vs. outlet) is taken, which is most often at the CT inlet [20]. The diameter ratio is calculated by measuring the diameter of the median nerve along its course within the CT (inlet to outlet). The maximum and minimum diameters are utilized to determine the ratio, which indicates the degree of compression within the CT. The WFR was calculated using the median nerve CSA at the CT inlet and at the mid-forearm.

The following diagnostic equipment was used in our study: EMG machine: UltraPro S100, Manufacturer: Natus Medical, Middleton, WI, USA, and Ultrasound: (1) Logq E and (2) P9, GE HealthCare, Chicago, IL, USA.

### 2.1. Inclusion and Exclusion Criteria

Inclusion criteria were patients 80 years and older with CTS confirmed by EDX studies. Of the patients referred to our Neurodiagnostic Center during the time period of this retrospective study, we included all patients aged 80 years and older with CTS who underwent a clinical examination, as well as EDX and US studies. Figure 1 features the flow chart of the study design. Patients with polyneuropathy or proximal median nerve neuropathies were excluded. Several metrics were collected including the patients’ gender and age, laterality (left/right/bilateral), diabetes mellitus, clinical examination (sensory decrease/loss, thenar atrophy, and weakness of the APB muscle), history of a carpal tunnel release (CTR) on the side of the symptoms, and severity of the median nerve entrapment at the CT by EDX findings. While numerous co-morbid conditions may contribute to CTS (autoimmune conditions, obesity, hypertension, and hyperlipidemia), we focused on diabetes mellitus in our study since this condition is a common co-morbid risk factor for CTS.

### 2.2. Statistical Analysis

Sensitivity, measured as true positives/(true positives + false negatives), was calculated for each US measurement, as well as combinations of measurements, in the diagnosis of CTS, with the EDX studies used as the gold standard. Each type of measurement was then tested as a predictor of the severity grade by EDX findings using a univariate ordinal regression. The distribution of EDX severity grade was compared between patients aged 40–50 years and those aged 80 years and older using a chi-squared test. Each US measurement (CSA at the CT inlet, WFR, and diameter ratio) was then tested as a predictor of the severity grade of EDX findings while controlling for diabetes mellitus using a multivariate ordinal regression. To correct for the multiple comparisons, a *p*-value ≤ 0.007 was considered statistically significant. All of these calculations were run on R Studio 4.2.3.

### 2.3. Institutional Review Board Approval of Research

Informed consent was obtained from all patients. All of the very elderly participants were able to sign the voluntary informed consent themselves. The University of Louisville IRB determined that our study was exempt under 45 CFR 46.101(b). The IRB number is 22.0873, and the Ethic Approval Code is 04092025. The IRB approval date was 9 April 2025.

## 3. Results

### 3.1. Demographics

A total of 187 patients aged 80 years and older were diagnosed with CTS by EDX studies over the course of this study (Table 1). The mean age was 84.0 years (range: 80–94 years), and the majority (107 [57.2%]) of patients were female. A higher number (102 [54.6%]) of patients had bilateral symptoms of CTS. A total of 174 (93.1%) patients were right-hand dominant, and the symptomatic side corresponded to hand dominance in 155 (82.9%) patients. Thirty-one (16.6%) patients had diabetes mellitus.

### 3.2. Clinical Findings

**Figure 2:**
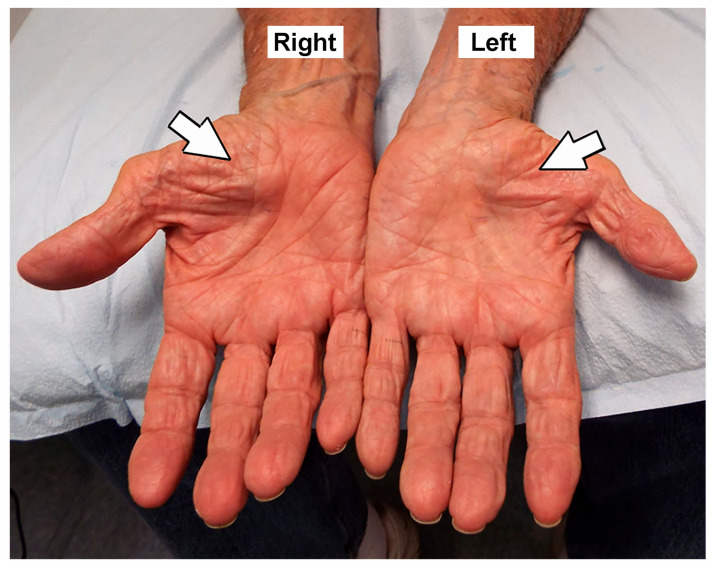
Palmar view showing bilateral thenar atrophy, more severe on the left. Left and right hands are identified.

The total number of very elderly hands with CTS was 289 (187 patients total, with bilateral symptoms in 102 patients) (Table 2). Of the 289 hands, thenar atrophy was observed in 75 (26.0%) hands (Figure 2), weakness of the APB muscle was detected in 178 (61.6%) hands, and pinprick loss was noted in 265 (91.7%) hands. Of the 289 hands, 120 (41.5%) had previously undergone a CTR.

### 3.3. Electrodiagnostic Studies

The severity of median nerve entrapment at the CT is highlighted in Table 3. Definitions of each level of severity (mild, moderate, moderately severe, and severe) are described in Table 3. Moderately severe and severe median nerve entrapment at the CT was seen in 118 (40.8%) and 134 (46.4%) hands, respectively. A total of 110 (38.1%) hands had non-localizing electrodiagnostic findings (Table 4). Seventy-five (68.2%) hands had no compound muscle action potentials (CMAP) or sensory nerve action potentials (SNAP), with a presumptive clinical and EDX diagnosis of severe CTS (Table 4).

Of the total 289 hands, 86 (29.8%) had no CMAP over the APB muscle (Table 5). A total of 57 (66.3%) hands had no CMAP over the APB and the second lumbrical muscles. The distal motor latency of the CMAP of the APB muscle was greater than 6.0 ms in 131 (45.3%) hands. The motor unit recruitment was decreased in 213 (73.7%) hands and was absent in 42 (14.5%) hands. Fibrillations and positive waves in the APB muscle were identified in 90 (31.1%) hands. SNAP was not detected in 211 (76.2%) hands (Table 6).

### 3.4. Ultrasound Studies

**Figure 3:**
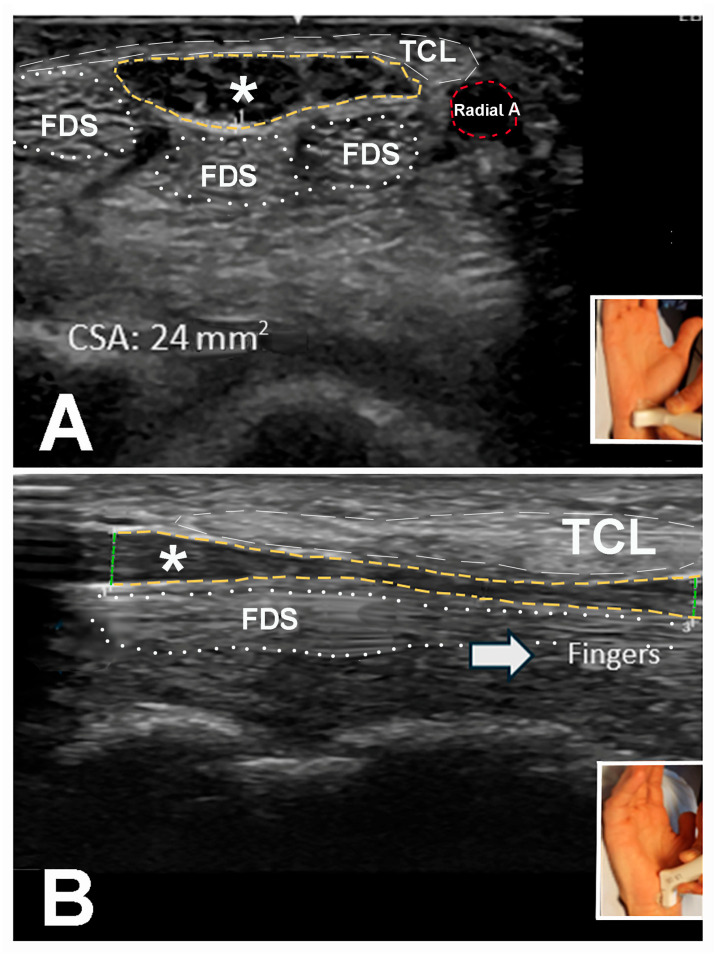
(**A**) Short-axis view of the median nerve at the carpal tunnel inlet with a cross-sectional area of 24 mm^2^ (normal: 10 mm^2^ or less). (**B**) Long-axis view of the median nerve within the carpal tunnel showing a decrease in the diameter of the median nerve within the carpal tunnel. The maximum/minimum diameter ratio is calculated from the maximum and the minimum diameter of the median nerve within the carpal tunnel. The insets indicate the positioning of the ultrasound probe for the long-axis and short-axis images of the median nerve. A single asterisk represents the median nerve. The green vertical line to the left of the asterisk in the long-axis view indicates the carpal tunnel inlet. The green vertical line to the right of the asterisk depicts the carpal tunnel outlet. Radial A: radial artery. TCL: transverse carpal ligament. FDS: flexor digitorum superficialis.

Of the 225 very elderly hands that underwent US studies, 183 (81.3%) hands had a CT inlet/outlet CSA between 10–20 mm^2^ (Table 7). Most (208 [92.4%)]) hands had a forearm CSA ≤ 10 mm^2^. The WFR was between 1.4 and 3.0 in 185 (82.2%) hands, and the minimum/maximum diameter ratio of the median nerve immediately proximal to the CT was between 0.5 and 0.75 in 129 (57.3%) and less than 0.5 in 92 (40.9%) hands (Figure 3A,B).

Comparing the sensitivities of various US measurements in diagnosing CTS in the very elderly, the CSA at the CT inlet had the highest sensitivity of the measurements studied at 100% (Table 8). The CSA at the CT inlet and the WFR were also tied for the highest sensitivity at 100% when diagnosing CTS in the very elderly with non-localizing EDX findings, as well as when these two measurements were used together (Table 9). As the CSA at the CT inlet increased, the odds of a greater CTS severity by EDX studies increased (OR = 1.109, *p*-value = 0.001) (Table 10). Additionally, as the minimum/maximum diameter ratio of the median nerve within the CT increases, the odds of a greater CTS severity decreased (OR = 0.045, *p*-value = 0.003).

**Figure 4:**
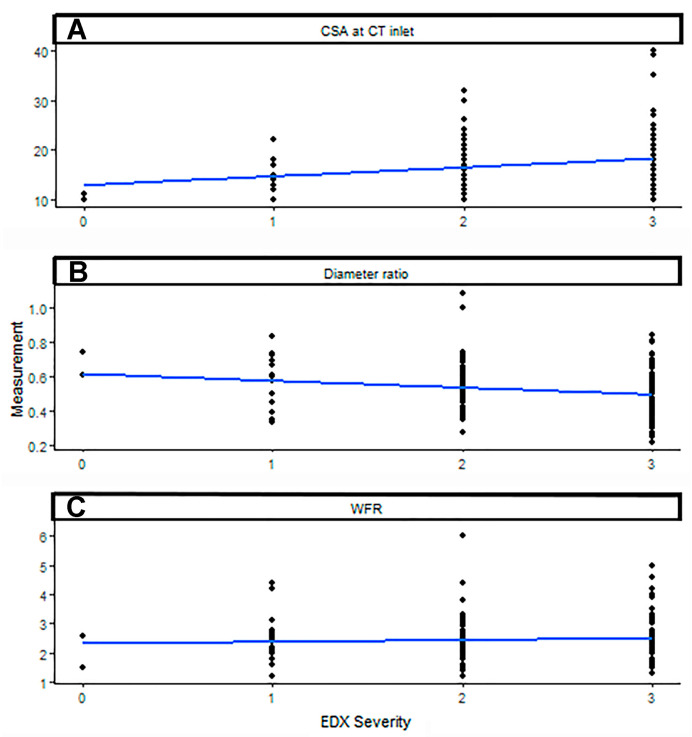
Ultrasound measurements of the (**A**) cross-sectional area of the carpal tunnel inlet, (**B**) minimum/maximum diameter ratio of the median nerve immediately proximal to the carpal tunnel, and (**C**) wrist–forearm ratio matched to carpal tunnel syndrome severity, with a regression line superimposed to show the relationship between these measurements and carpal tunnel syndrome severity. CSA: cross-sectional area; CT inlet: carpal tunnel inlet; diameter ratio: minimum/maximum diameter ratio of the median nerve immediately proximal to the CT; WFR: wrist–forearm ratio; EDX: electrodiagnostic.

**Figure 5:**
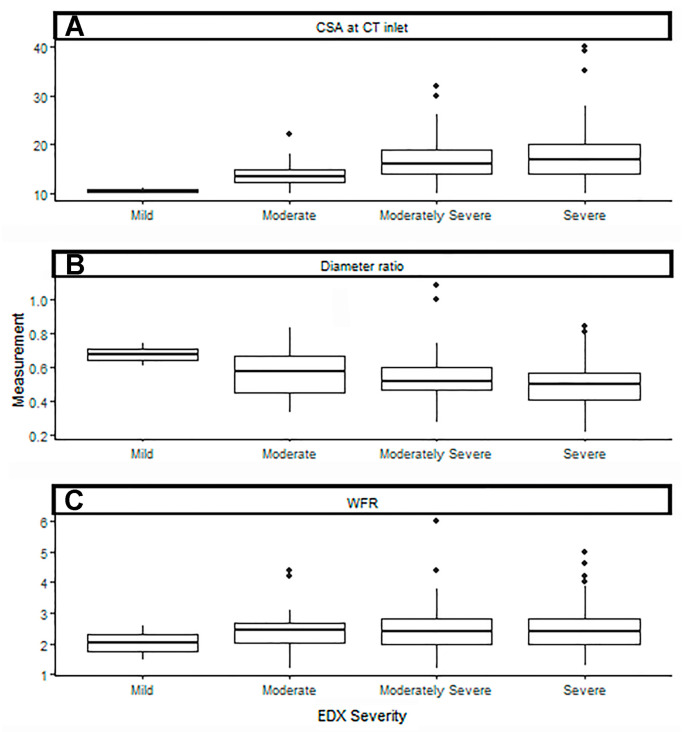
The spread of ultrasound measurements of the (**A**) cross-sectional area of the carpal tunnel inlet, (**B**) minimum/maximum diameter ratio of the median nerve immediately proximal to the carpal tunnel, and (**C**) wrist–forearm ratio for each carpal tunnel syndrome severity level. CSA: cross-sectional area; CT inlet: carpal tunnel inlet; diameter ratio: minimum/maximum diameter ratio of the median nerve immediately proximal to the CT; WFR: wrist–forearm ratio; EDX: electrodiagnostic.

The association between US measurements and the severity of CTS by EDX studies was analyzed (Table 11). As the CSA at the CT inlet increased, the CTS severity increased (*p*-value = 0.001). As the minimum/maximum diameter ratio of the median nerve immediately proximal to the CT decreased, the CTS severity also increased (*p*-value = 0.02). Figure 4 depicts the US measurements matched to CTS severity, with a regression line superimposed to show the relationship between these measurements and CTS severity. The spread of US measurements is shown for each CTS severity level (Figure 5). When controlling for diabetes mellitus, none of our US measurements were able to predict CTS at a significant level (Supplemental Table S1).

### 3.5. Comparison of the Severity of Carpal Tunnel Syndrome by Electrodiagnostic Studies Between Two Age Cohorts

Between 1 January 2024 and 31 December 2024, there were 81 hands diagnosed with CTS by EDX studies in patients aged 80 years and older at our Neurodiagnostic Center and 127 hands diagnosed with CTS by EDX studies in patients aged 40–50 years. Of the 81 very elderly hands, 42 (51.8%) had severe CTS and 2 (2.5%) had mild CTS by EDX studies (Table 12). Contrarily, of the 127 hands in the middle age group (40–50 years) in the same year, 2 (1.6%) had severe CTS and 60 (47.2%) had mild CTS. The very elderly patients with CTS more frequently had more severe CTS than the middle-aged patients with CTS (chi-squared = 102.65_3_, *p*-value < 0.001). The power for a chi-squared test of 208 subjects in eight categories (the two age groups in the four severities) was 0.99.

## 4. Discussion

Only a few studies have assessed older patients with CTS to determine whether any particular features are unique to this population [9,11,12,13,14]. In Blumenthal and colleagues’ study of 343 patients with CTS, 158 were young (≤50 years), 115 were middle-aged (51–64 years), and 70 were elderly (≥65 years) [12]. The elderly had a higher prevalence of thenar weakness and atrophy and more severe median nerve entrapment compared to younger patients. The elderly also had more prolonged latencies, reduced response amplitudes, and slowed conduction velocities in the median motor and sensory responses. Additionally, more elderly patients had absent SNAP. These authors stress the importance of objective evidence of CTS severity in the elderly as opposed to subjective complaints [12]. In Seror’s study comparing elderly (>70 years) versus middle-aged (50–60 years) patients with CTS, 60% of the elderly group had severe electrophysiological motor and sensory denervation of CTS compared to only 18% of the middle-aged group [14]. Interestingly, 18% of the elderly cohort were asymptomatic, although 25% had severe neurological impairment. To avoid permanent disability, these authors stress that the elderly should be regarded as a high-risk group of severe cases [14]. In Finger and colleagues’ study of 295 patients with CTS, divided into three age groups (28 years and younger [23 hands], 29–71 years [248 hands], and 72 years and older [24 hands]), the overall accuracy for US and nerve conduction studies (NCS) was 66% for both tests when evaluating all age groups [11]. In the oldest group, the NCS showed a 94% sensitivity and a 25% specificity compared to an 81% sensitivity and 38% specificity for US. These authors conclude that there is a substantial inaccuracy with both tests compared to a validated clinical diagnostic tool such as the CTS symptoms scale (CTS-6) as the reference standard [11].

Several measurements of the median nerve CSA may prove useful in determining the severity of CTS by US, especially in elderly patients [5,6,9]. Roghani and colleagues’ study of elderly (>60 years) patients with CTS who underwent US, the sensitivity and specificity of the CSA inlet were 96.9% and 93.6%, respectively, and 99% and 28% for the CT inlet–antecubital CSA ratio, respectively [6]. In Miwa and colleagues’ study of 279 hands of patients with CTS and 50 normal hands, the CSA correlated with distal motor and sensory latencies of the median nerve and severity of CTS [5]. CTS severity increased with age, especially in patients over the age of 80; however, their median nerve CSA was not enlarged despite their high severity of CTS. These authors emphasize that the diagnostic importance of median nerve CSA may be limited in elderly patients [5]. Mulroy and colleagues corroborate Miwa et al.’s findings that median nerve CSA at the wrist is not a sensitive marker of CTS in the very elderly [9]. In Mulroy et al.’s study of 92 patients in two age groups (40–65 years and 80–95 years) with CTS, US was less sensitive in the elderly versus the younger groups (54% vs. 87%) and did not correlate with clinical or EDX severity [9]. Compared to the younger cohort, the elderly patients’ CTS was both clinically and electrodiagnostically more severe. In their study, the maximal CSA at the wrist was significantly larger in younger patients. Most very elderly patients with severe CTS showed no significant median nerve enlargement, and there was no association between the median nerve CSA and CTS severity. These authors attributed the absence of median nerve enlargement at the wrist by US in the very elderly to these patients’ experiencing the final stages of chronic median nerve injury marked by nerve atrophy and fibrotic changes [9]. Furthermore, the very elderly may respond to chronic nerve compression differently from a pathophysiological standpoint compared with younger patients with CTS. Our study is comparable to that of Mulroy et al., with similar age groups and the use of EDX and US studies in the evaluation of CTS. We concur that the very elderly population has a greater severity of CTS compared to the middle-aged group. Our study had a larger number of very elderly hands with CTS over a longer period of time than Mulroy et al.’s work (289 hands over 6 years vs. 40 hands over 1 year, respectively). Unlike Mulroy and Pelosi’s study, as the CSA at the CT inlet increased in our study, the odds of a greater CTS severity by EDX studies increased.

US served an important role in our study since almost 40% of hands in the very elderly group had non-localizing EDX findings in severe CTS. In patients with a total loss of SNAP and CMAP as seen in severe CTS, the EDX localization of the median nerve neuropathy is not conclusive. A similar issue arises with retrograde slowing of motor conduction, leading to decreased motor conduction velocity in the forearm. Under these circumstances, the distinction between a lesion of the median nerve at the CT and a lesion in the forearm distal to the origin of the AIN branch is difficult. This type of presentation is common in severe median nerve neuropathy at the CT in the very elderly. US can readily confirm the location of the median nerve neuropathy when EDX studies are non-localizing. In our previous US study of severe distal median nerve neuropathy in 46 patients, 42 had severe entrapment at the CT, and 4 had more proximal lesions [21].

The findings in the present study raise the question of whether US should be the preferred test for the evaluation of CTS in the very elderly, as the majority of these patients present with features of severe CTS. As axon loss may be greater in this population, there may be a need for different cut-off values for CSA evaluations by US. In our work, the CSA at the CT inlet had the highest sensitivity of the US measurements studied for diagnosing CTS. Both CSA at the CT inlet and the WFR were equal in sensitivity for diagnosing CTS in the very elderly with non-localizing EDX findings. Our results suggest that CSA may increase significantly in very elderly patients with CTS as well. More advanced US imaging techniques, such as quantitative ultrasound (QUS), US elastography, and Superb microvascular imaging (SMI), may provide more insight into the underlying mechanism of nerve enlargement in different age groups.

The EDX findings were the criteria we used for confirming the diagnosis of CTS and to grade its severity; however, there were exceptions. One such exception occurred when no SNAPs or CMAPs of the APB and second lumbrical muscles were recordable, resulting in a “non-localizing” study. The needle EMG abnormality may still be useful, but it is not as precise, as it may not differentiate involvement at the distal forearm from entrapment at the CT. This situation highlights the advantage of US over EDX tests in certain circumstances.

Several theories have been proposed to explain the apparent increase in CTS in the elderly, including better medical attention to the elderly, increased life span in western countries, improved knowledge of CTS by physicians, and enhanced reliability on EDX studies to diagnose this condition [12]. It has also been suggested that the more severe findings on EDX studies in the elderly may be attributed to delayed treatment due to age-related reduced pain sensitivity because of decreased nerve membrane excitability [22,23]. The elderly may also ignore symptoms until activities like buttoning and zipping are difficult. Additionally, primary care physicians may not ask questions to identify CTS early and often miss the thenar atrophy.

The treatment of CTS in elderly patients has proven challenging due to an unpredictable response to CTR. Several studies have analyzed the outcomes of CTR in older patients [15,16,17,24,25,26,27,28]. In Fung et al.’s review of CTR in the elderly (≥65 years) and Hobby et al.’s study of surgical outcomes following CTR, elderly patients are less satisfied postoperatively compared to younger patients [16,25]. The elderly also had less predictable symptomatic and functional improvement after a CTR compared to younger patients. In Stone and colleagues’ study of CTR in two age groups (97 patients aged 80 years and older and 659 patients less than 80 years), the super-elderly patients were more likely to present with thenar muscle atrophy and have a severe conduction deficit compared with younger patients [15]. The functional outcome and satisfaction rates were similar between the two age groups. Due to the more severe presentation of elderly patients with CTS, both Fung et al. and Stone et al. recommend an earlier CTR [15,16].

In Ko and colleagues’ study of 304 wrists that underwent an endoscopic CTR (including 48 in patients aged > 70 years), the elderly group had more severe clinical symptoms, a higher frequency of thenar atrophy, NCS grades showing more severe disease, and more prominent median nerve swelling [17]. Additionally, elderly patients had poorer outcomes postoperatively than younger patients. In Porter et al.’s study of 87 patients who completed a validated self-administered questionnaire following a CTR, improvement in symptoms and function decreased with increasing age, which was most notable in patients over 60 years of age [28]. The level of satisfaction postoperatively was also lower in the elderly compared to younger patients. In Zyluk and Puchalski’s study of 386 patients who underwent a CTR, divided into three age groups (28 patients 40 years and younger, 248 patients aged 41–64 years, and 73 patients older than 65 years), all patients had significant resolution of symptoms at 6-month follow-up [27]. However, patients older than 60 years demonstrated less improvement in strength. In Hansen and Larsen’s study of 101 patients aged 23–94 years who underwent an endoscopic CTR, patients older than 65 years had a less favorable short-term outcome [26].

### Strengths and Limitations

The strength of the present study is that it features the largest number of very elderly patients with CTS who underwent EDX and US studies. Limitations of this study include its retrospective nature and lack of follow-up, as most patients were only evaluated on one occasion at our Neurodiagnostic Center. We were unable to evaluate whether very elderly patients underwent a CTR and whether they were satisfied and had attained symptom improvement postoperatively. In this study, we considered 2024 as the representative year of previous years for our middle-aged group (40–50 years) of patients with CTS. However, it is a limitation of our work that we only selected 1 year to be representative. While several co-morbid conditions may contribute to CTS (autoimmune conditions, obesity, hypertension, and hyperlipidemia), we only focused on diabetes mellitus, which is a limitation of our study. Quantified clinical measures like the Boston Carpal Tunnel Syndrome Questionnaire (BCTQ) were not performed in our study, as the goal was to confirm and grade the size of the median nerve entrapment at the carpal tunnel by EDX and US tests. We documented the presence of thenar muscle weakness/atrophy and sensory loss in the median nerve distribution in every patient. A limitation of the current study is that the EDX severity for the middle-aged patients (ages 40–50 years) was based on diagnostic coding. Another limitation is that we described only the EDX severity for the middle-aged patients and not other clinical characteristics. The goal of the present study was to describe the characteristics of CTS in the very elderly, and not a detailed comparison study between the two age cohorts.

## 5. Conclusions

In our study, very elderly patients present with more severe CTS compared to middle-aged patients based on EDX studies. Primary care physicians should look for signs and symptoms of CTS in the very elderly as part of a routine check-up to detect CTS early. US is highly useful in confirming CTS when the EDX studies are non-localizing, as in severe CTS, frequently observed in the very elderly cohort.

## Figures and Tables

**Table 1:** Clinical findings of carpal tunnel syndrome in the very elderly.

Characteristics	Categorization	Total Number of Patients (n = 187)
Age (mean)	80–84 years 85–89 90–94 years	84 years (80–94 years) 106 (56.7%) 68 (36.4%) 13 (6.9%)
Sex	Male Female	80 (42.8%) 107 (57.2%)
Side of symptoms	Left only Right only Bilateral	33 (17.6%) 52 (27.8%) 102 (54.6%)
Hand dominance	Left Right Ambidextrous	10 (5.3%) 174 (93.1%) 3 (1.6%)
The side of symptoms corresponded to hand dominance	Yes No	155 (82.9%) 32 (17.1%)
Diabetes mellitus	Yes No	31 (16.6%) 156 (83.4%)

**Table 2:** Neurological examination in the very elderly (total number of hands).

Characteristics	Total Number of Hands (n = 289)
Thenar atrophy	75 (26.0%)
Weakness of the APB muscle	178 (61.6%)
Loss of pinprick sensation	265 (91.7%)
History of carpal tunnel release on side of symptoms	120 (41.5%)

APB: abductor pollicis brevis.

**Table 3:** Severity of median nerve entrapment at the carpal tunnel by electrodiagnostic findings in the very elderly (total number of hands).

Severity	Criteria	Total Number of Hands (n = 289)
Mild	Only sensory fascicles affected	3 (1.0%)
Moderate	Sensory and motor fascicles affected	34 (11.8%)
Moderately severe	Sensory and motor fascicles affected with motor unit changes (increase in polyphasic units) in APB	118 (40.8%)
Severe	Loss of SNAP + loss of or decrease in amplitude CMAP of APB < 1 mV, along with needle EMG showing denervation of APB	134 (46.4%)

APB: abductor pollicis brevis; SNAP: sensory nerve action potential; CMAP: compound muscle action potential.

**Table 4:** Non-localizing electrodiagnostic findings in the very elderly (total number of hands).

Electrodiagnostic Findings	Total Number of Hands (n = 110)
No CMAP or SNAP (presumptive clinical and EDX diagnosis of severe CTS)	75 (68.2%)
Slow motor conduction velocity proximal to wrist (retrograde slowing)	28 (25.4%)
Dissimilar CMAP on proximal stimulation (Martin Gruber communication)	6 (5.4%)
Patient unable to tolerate proximal stimulation	1 (1.0%)

CMAP: compound muscle action potential; SNAP: sensory nerve action potential; CTS: carpal tunnel syndrome; EDX: electrodiagnostic.

**Table 5:** Details of motor electrodiagnostic findings in carpal tunnel syndrome in the very elderly (total number of hands).

Motor Electrodiagnostic Findings	Total Number of Hands (n = 289)
No CMAP over APB	86 (29.8%)
CMAP of APB > 4 mV	46 (15.9%)
CMAP of APB 2–4 mV	76 (26.3%)
CMAP of APB 1–2 mV	39 (13.5%)
CMAP of APB < 1 mV	42 (14.5%)
No CMAP over APB and 2nd lumbrical (n = 86)	57 (66.3%)
No CMAP over APB with measurable CMAP over 2nd Lumbrical (n = 86)	29 (33.7%)
CMAP of 2nd lumbrical > 2 mV	0 (0%)
CMAP of 2nd lumbrical < 2 mV	29 (33.7%)
Distal motor latency to APB > 6.0 ms	131 (45.3%)
Distal motor latency to APB 3.7–6.0 ms	72 (24.9%)
Normal motor unit morphology	39 (13.5%)
Increase in polyphasic motor units	207 (71.6%)
Normal motor unit recruitment	34 (11.8%)
Decrease in motor unit recruitment	213 (73.7%)
No motor units	42 (14.5%)
Fibrillations and positive waves in APB	90 (31.1%)

APB: abductor pollicis brevis; CMAP: compound muscle action potential.

**Table 6:** Details of sensory electrodiagnostic findings in carpal tunnel syndrome in the very elderly (total number of hands).

Sensory Electrodiagnostic Findings	Total Number of Hands (n = 277)
No SNAP	211 (76.2%)
SNAP amplitude > 25 uV	5 (1.8%)
SNAP amplitude 10–25 uV	31 (11.2%)
SNAP amplitude < 10 uV	30 (10.8%)
SNAP latency > 6.0 ms	3 (1.1%)
SNAP latency 3.3–6 ms	63 (22.7%)

SNAP: sensory nerve action potential.

**Table 7:** Ultrasound findings of the median nerve in the very elderly (total number of hands).

Ultrasound Findings	Total Number of Hands (n = 225)
CT inlet/outlet CSA 10–20 mm^2^	184 (81.8%)
CT inlet/outlet CSA > 20 mm^2^	41 (18.2%)
Forearm CSA > 10 mm^2^	17 (7.6%)
Forearm CSA ≤ 10 mm^2^	208 (92.4%)
W/F ratio < 1.4	4 (1.8%)
W/F ratio 1.4–3.0	185 (82.2%)
W/F ratio > 3.0	36 (16.0%)
Diameter min/max ratio > 0.75	4 (1.8%)
Diameter min/max ratio 0.5–0.75	129 (57.3%)
Diameter min/max ratio < 0.5	92 (40.9%)

CT: carpal tunnel; CSA: cross-sectional area; W/F: wrist/forearm; min/max: minimum maximum.

**Table 8:** Sensitivity of various ultrasound measurements in diagnosing carpal tunnel syndrome in the very elderly.

Ultrasound Measurements	Sensitivity
CSA at CT inlet	1
WFR	0.991
Diameter ratio	0.973
All three measures	0.964
CSA and WFR	0.991
CSA and diameter ratio	0.973

CSA: cross-sectional area; WFR: wrist–forearm ratio; Min/max diameter ratio: minimum/maximum diameter ratio of the median nerve within the carpal tunnel.

**Table 9:** Sensitivity of various ultrasound measurements in diagnosing carpal tunnel syndrome in the very elderly with non-localizing electrodiagnostic findings.

Ultrasound Measurements	Sensitivity
CSA at CT inlet	1
WFR	1
Diameter ratio	0.968
All three measures	0.968
CSA and WFR	1
CSA and diameter ratio	0.968

CSA: cross-sectional area; WFR: wrist–forearm ratio; min/max diameter ratio: minimum/maximum diameter ratio of the median nerve within the carpal tunnel.

**Table 10:** Univariate Ordinal Regression: Finding the ability of each measurement to predict the severity of a very elderly patient’s electrodiagnostic findings.

Ultrasound Measurements	OR	2.50%	97.50%	*p*-Value
CSA at CT inlet	1.109	1.045	1.183	0.001
WFR	1.186	0.828	1.722	0.359
Diameter Ratio	0.045	0.006	0.337	0.003

OR: odds ratio; CSA: cross-sectional area; WFR: wrist–forearm ratio; min/max diameter ratio: minimum/maximum diameter ratio of the median nerve within the carpal tunnel.

**Table 11:** Associations between ultrasound measurements and severity of carpal tunnel syndrome by electrodiagnostic studies in the very elderly.

Ultrasound Measurements	Mild	Moderate	Moderately Severe	Severe	*p*-Value
n	2	18	89	111	
CSA at CT inlet (median [IQR])	10.50 [10.25, 10.75]	13.50 [12.25, 15.00]	16.00 [14.00, 19.00]	17.00 [14.00, 20.00]	0.001
WFR (median [IQR])	2.05 [1.77, 2.33]	2.45 [2.02, 2.70]	2.40 [2.00, 2.80]	2.40 [2.00, 2.80]	0.651
Diameter ratio (median [IQR])	0.67 [0.64, 0.71]	0.58 [0.45, 0.67]	0.52 [0.47, 0.60]	0.50 [0.41, 0.57]	0.02

CSA: cross-sectional area; WFR: wrist–forearm ratio; min/max diameter ratio: minimum/maximum diameter ratio of the median nerve within the carpal tunnel; IQR: interquartile range.

**Table 12:** Comparison of the severity of carpal tunnel syndrome by electrodiagnostic findings between the very elderly and middle-aged (total number of hands) cohorts in 2024.

EDX Severity of Carpal Tunnel Syndrome	Middle Age (Ages 40–50 Years) (n = 127)	Very Elderly (80 Years and Older) (n = 81)
Mild	60 (47.2%)	2 (2.5%)
Moderate	39 (30.7%)	10 (12.3%)
Moderately severe	26 (20.5%)	27 (33.3%)
Severe	2 (1.6%)	42 (51.8%)

## Data Availability

All of the data for this study is included in the current article.

## Supplementary Materials

The following supporting information can be downloaded at: https://www.mdpi.com/article/10.3390/neurolint17090137/s1, Table S1: Multinomial ordinal regression judging the ability of ultrasound measures at predicting carpal tunnel syndrome, controlling for diabetes mellitus.
