# Supplementary material for: Carpal Tunnel Syndrome in the Very Elderly: Clinical, Electrodiagnostic, and Ultrasound Features in a Cohort of 187 Patients
Source: Neurol Int. 2025 Aug 30;17(9):137. doi: 10.3390/neurolint17090137 (PMC12472666; doi:10.3390/neurolint17090137)
Supplement: Supplementary file 1 [file neurolint-17-00137-s001.zip › neurolint-3776277-supplementary.pdf]

Supplemental Table S1: Multinomial Ordinal Regression Judging the Ability of Ultrasound Measures at Predicting Carpal Tunnel Syndrome, Controlling for Diabetes Mellitus

Regression	Variables	OR	2.50%	97.50%	P-Value
**CSA at CT inlet Regression**	DM	0.876	0.392	1.983	0.748
	CSA at CT inlet	1.103	1.028	1.193	0.010
**WFR Regression**	DM	0.843	0.380	1.888	0.674
	WFR	1.120	0.693	1.836	0.646
**Diameter Ratio Regression**	DM	0.812	0.365	1.821	0.609
	Diameter ratio	0.130	0.009	1.692	0.122

OR: odds ratio

DM: diabetes mellitus

CSA: carpal tunnel syndrome

CT: carpal tunnel

WFR: wrist-forearm ratio
